# Estimation of the Long-Term Care Needs of Stroke Patients by Integrating Functional Disability and Survival

**DOI:** 10.1371/journal.pone.0075605

**Published:** 2013-10-04

**Authors:** Mei-Chuan Hung, Ching-Lin Hsieh, Jing-Shiang Hwang, Jiann-Shing Jeng, Jung-Der Wang

**Affiliations:** 1 Department of Public Health, National Cheng Kung University College of Medicine, Tainan, Taiwan; 2 School of Occupational Therapy, College of Medicine, National Taiwan University, and Department of Physical Medicine and Rehabilitation, National Taiwan University Hospital, Taipei, Taiwan; 3 Institute of Statistical Science, Academia Sinica, Taipei, Taiwan; 4 Stroke Center and Department of Neurology, National Taiwan University Hospital, Taipei, Taiwan; 5 Departments of Internal Medicine and Occupational and Environmental Medicine, National Cheng Kung University Hospital, Tainan, Taiwan; Kaohsiung Chang Gung Memorial Hospital, Taiwan

## Abstract

**Objectives:**

This study aimed to estimate the dynamic changes of different physical functional disabilities and life-time care needs for patients with stroke.

**Data Sources and Study Design:**

We examined a hospital-based cohort including 16,043 patients who had their first stroke during 1995–2010. The Barthel Index (BI) was used to measure disability levels in 1,162 consecutive patients, with a total of 1,294 measurements at the stroke clinics and the rehabilitation wards, and a cross-sectional design.

**Extraction Methods:**

The survival function was extrapolated to lifetime by a semi-parametric method and multiplied with proportions of different disabilities over time to obtain the long-term care needs for different stroke subtypes.

**Principal Findings:**

On average, stroke patients would suffer at least 0.86 years with mild disability, 1.24 years with moderate disability and 1.39 years with severe disability, as measured by the BI. Among these, patients with a cardio-embolic infarct or intracerebral hemorrhage (ICH) suffered more than 2 years of severe disability. Assistance in bathing was the most common need for care in stroke patients.

**Conclusions:**

Among different subtypes of stroke, cardio-embolic infarct and ICH lead to the longest durations of severe physical functional disability. The method presented in this work may also be applied to other chronic diseases and different functional disabilities.

## Introduction

Worldwide, stroke is the second leading cause of death [1]. In the United States alone, there are 795,000 patients with new or recurrent strokes and approximately 128,900 stroke-related deaths every year. There are also more than seven million stroke survivors, who cost the US economy about $38.6 billion per year [2]. Although current data show that more than 32% of these patients have received outpatient rehabilitation [2], the lifelong need for care of stroke patients with disabilities has not been fully explored [3].

Stroke is a highly heterogeneous disorder with distinct subtypes, each of which presents specific clinical pictures [2], [4]–[7], including a range of functional disabilities [8]–[10]. Information on the dynamic changes of functional disabilities for different stroke subtypes is useful to improve the quality of clinical management, rehabilitation, and long-term care. Furthermore, quantification of the length of different disability levels can be used to project potential long-term care needs, as well as profile changes in these levels. However, because a systematic collection of such data from established patient cohorts has been lacking, we conducted this study to quantify dynamic time trends in different physical functional disability states using the Barthel Index (BI) and to estimate life-time care needs for patients with different subtypes of stroke. As BI has a well recognized floor and ceiling effect [11], [12], and does not include performance other than physical function, this effort could be regarded as the beginning of more extensive assessments of other functional disabilities, such as cognitive ones, as well as of the care needed by such patients.

## Methods

### Establishing the Stroke Patient Cohort

The study was approved by the Institutional Review Board of the National Taiwan University Hospital (NTUH) (IRB number: 9561703047) prior to commencement, and every interviewed patient provided written informed consent. We used data from the stroke registry of the NTUH [7], [13], which was established in 1995 in order to investigate the etiological factors, clinical courses, and outcomes of stroke. At the time of this study, the registry contained information on 16,043 patients who experienced their first stroke between 1995 and 2010. The patients were categorized into four groups: intracerebral hemorrhage (ICH), large artery atherothrombotic infarct (LAA), lacunar infarct, cardio-embolic infarct (CEI), and infarct of other specific or undetermined etiologies. The diagnosis of ICH was made when a parenchymal hemorrhage, as identified through brain imaging, corresponded to clinical observations. Subarachnoid hemorrhage was not included in this study [7], [13]. The diagnostic criteria for ischemic stroke were adopted from the Trial of Org 10172 in Acute Stroke Treatment (TOAST) classification system [4].

### Definition of Disability Levels and Collection of Cross-sectional Data

From September 2008 to August 2012, patients from the stroke clinics of the NTUH with ischemic stroke or ICH were invited for functional and questionnaire assessments using a cross-sectional design. Patients were not recruited if they had disturbances in consciousness, moderate to severe dementia or aphasia, or an inability to appropriately communicate with the clinicians. To broaden the study sample, we also included consecutive patients with strokes who were hospitalized in the rehabilitation wards. The patients were assessed on the date of discharge between January 2009 and August 2012. Some of these patients were assessed again one year after the initial diagnosis through telephone interviews. Each subject was assessed by the investigator and/or research assistants who were formally trained in administering the BI and the related assessments [14].

The BI is composed of 10 items with varying weights, and has been shown to have good reliability and validity [11], [12]. Two items regarding grooming and bathing are assessed using a two-point scale (0 and 5 points); six items regarding feeding, toilet use, ascending and descending stairs, dressing, controlling bowels, and bladder control are scored on a three-point scale (0, 5 and 10 points), and two items regarding moving from a wheelchair to bed and returning and walking on a level surface are evaluated on a four-point scale (0, 5, 10, and 15 points). The total score can range from 0 to 100, with higher scores reflecting more independence and lower scores representing greater dependency. The BI scores were classified into four categories: no disability (BI = 100), mild disability (BI: 90–95), moderate disability (BI: 60–85) and severe disability (BI: ≤55) [15], [16]. We also used the original score of each item to estimate detailed long-term care needs in stroke patients.

### Survival Analysis and Extrapolation

The survival status of all the registered stroke patients was determined by cross-linkage with the National Mortality Registry in Taiwan. The patients were followed from the time that they were diagnosed with a stroke until they were deceased or censored on December 31, 2010. We applied the Kaplan-Meier method to estimate survival for different stroke subtypes from the onset of stroke. However, survival can only be estimated through the time limit of follow-up with this method [17], whereas many young stroke patients in our cohort may survive for more than 20 or 30 years. We thus employed a semi-parametric extrapolation method, which only requires an assumption of constant excess hazards or mortality to estimate lifelong survival [18]. The estimates were obtained using iSQoL software [19]. The feasibility and accuracy of the methods and software used in this work have been demonstrated in cohorts with stroke [13] and different cancers [20], [21], and the relative biases for the estimates of extrapolation are usually within 5–10% [20], [21]. The semi-parametric method can produce accurate survival extrapolations, because it uses additional information from age- and sex-matched referents, and the logit transformation of the survival ratio between patients and referents has been shown to be linear over time [22]. We also estimated the expected years of life loss (EYLL), which can be obtained by subtracting the area under the survival curve of stroke patients after diagnosis from that of the sex- and age-matched reference population in our study [22]. Detailed methods and mathematical proofs are described in our previous reports [17]–[23].

### Estimation of Lifelong Duration for Different Disability Levels and Long-term Care

The health status of a subject with a specific condition can be classified into *k* exclusive categories denoted as 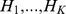 according to a given measure. The function 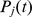 can be interpreted as the proportion of the surviving subjects whose health statuses are 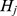 at time *t*, of which the sum of 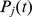 is one. The estimate of mean lifelong duration 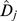 of the population with health status 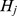 can thus be obtained by multiplying the estimates of survival probability 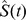 by the proportion 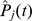 of the specific health status at time *t*, and then summing up throughout the patient’s lifetime, i.e.,
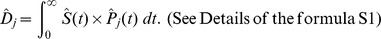



In this study, we applied a kernel smoothing method of averaging the nearest 10% of the observed proportions of living subjects with health status 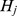 around time *t* to obtain 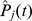 throughout the period from diagnosis to interview (or, duration-to-date) [23], of which the maximum in this study was 26 years. This approach can capture the proportion of patients with a specific level of disability among those who survive up to a specific length of time, and can be represented by those surveyed cross-sectionally, if it is a random sample and the sample size exceeds 50 [23]. Given the analysis based on the BI total score, the health status was classified into four exclusive categories: disability-free 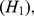 mild disability 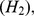 moderate disability 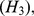 and severe disability 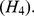 The expected years of life living with disability (EYLDs) can be estimated by subtracting the lifelong duration with the disability-free estimate, 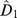, from the estimate of the life expectancy of the population, which is equal to 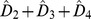. The estimates of the lifelong duration of disability can be obtained using the free iSQoL software (iSQoL 2013) [19]. Similarly, we can obtain estimates of the lifelong duration of disability levels for each item measured in the BI. For example, the functional evaluation of bathing in a stroke patient was classified into two exclusive categories of score 5 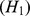 and score 0 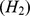, and we can estimate the lifetime duration of score 0 for patients with different stroke subtypes. The confidence limits of the above estimations were calculated by a bootstrap method, and z-tests were also performed, with a p-value <.05 considered statistically significant.

### Other Statistical Analysis

The differences in the frequency distributions of stroke patients between the NTUH and the cross-sectional sample at the stroke clinics and rehabilitation wards were tested with the Chi-squared test, and *p*<0.05 was regarded as significant. The analysis was carried out using SAS (ver.9.2) software.

## Results

**Figure 1:**
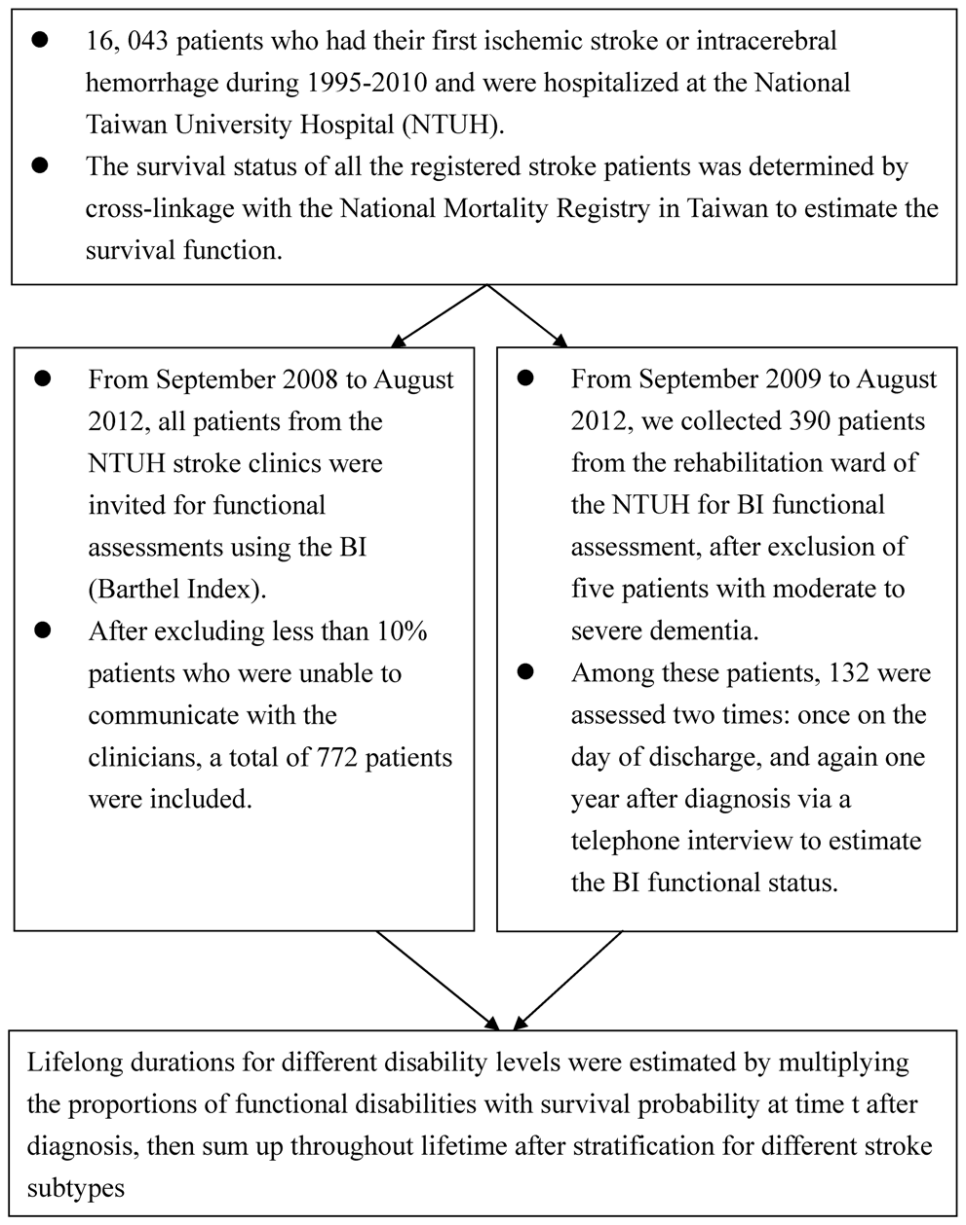
Flow chart of the computation process for lifelong duration of different disability levels and long-term care.

Because the NTUH Stroke Center has gained the trust of patients during its 15 years of operation, none of the eligible patients refused to be assessed, except for those who were totally unable to communicate with the clinicians. The non-respondents at the stroke clinics were less than 10% of the invited patients, while only five patients with moderate to severe dementia could not be performed BI assessment at the rehabilitation ward (Figure 1). The median duration from onset to the date of interview was 12.9 months (range, 0.1–318.8 months). Among these, 132 patients recruited from the rehabilitation ward were assessed two times: once on the day of discharge, and again one year after diagnosis via a telephone interview. A comparison of the frequency distributions of the patients’ demographic and clinical characteristics is given in Table 1, which shows that the age and diabetes distributions of the smaller sample interviewed at the clinic and bedside seem similar to those of the larger cohort at the NTUH Stroke Center, as well as those abstracted from registered inpatients in the National Health Insurance (NHI) database. The cross-sectional sample was composed of slightly lower proportions of patients with cancer or ischemic heart disease, and thus the prevalence of functional disabilities may be underestimated. Although the interviewed sample is composed of a higher proportion of ischemic stroke and lacunar infarct patients, all the sample sizes of different stroke subtypes are more than 169, which is sufficiently large for estimation of changes in functional disabilities with different durations-to-date.

**Table 1:** Comparison of frequency distributions of stroke patients hospitalized under the National Health Insurance (NHI), at the National Taiwan University Hospital (NTUH), and a cross-sectional sample at the stroke clinics and rehabilitation wards.

	NHI	NTUH	Cross-sectional sample
Case number	79,816	16,043	1,294
Calendar years of data collection	2008–2009	1995–2010	2008–2012
Sex (% male)*	59.6	59.3	65.2
Age in years, mean(SD)†	66.9 (14.2)	65.1 (15.0)	65.7 (12.6)
Types of stroke			
Infarct (%)*	62,111 (77.8)	12,397 (77.3)	1,097 (84.8)
Large artery atherothrombosis	–	2,329 (14.5)	292 (22.6)
Lacune	–	3,702 (23.1)	360 (27.8)
Cardio-embolism	–	2,504 (15.6)	169 (13.1)
Other	–	3,862 (24.1)	276 (21.3)
Intracerebral hemorrhage (%)*	17,705 (22.2)	3,646 (22.7)	197 (15.2)
Comorbidities (%)			
Hypertension*	59,696 (75.1)	11,460 (71.4)	1,053 (81.4)
Diabetes	31,041 (38.9)	5,072 (31.6)	418 (32.3)
Atrial fibrillation	7,876 (9.9)	2,525 (15.7)	179 (13.9)
Ischaemic heart disease*	9,093 (11.4)	2,532 (15.8)	104 (8.4)
Cancer*	6,097 (7.6)	1,921 (12.0)	61 (4.7)

* P<0.001;

† SD: standard deviation.

**Figure 2:**
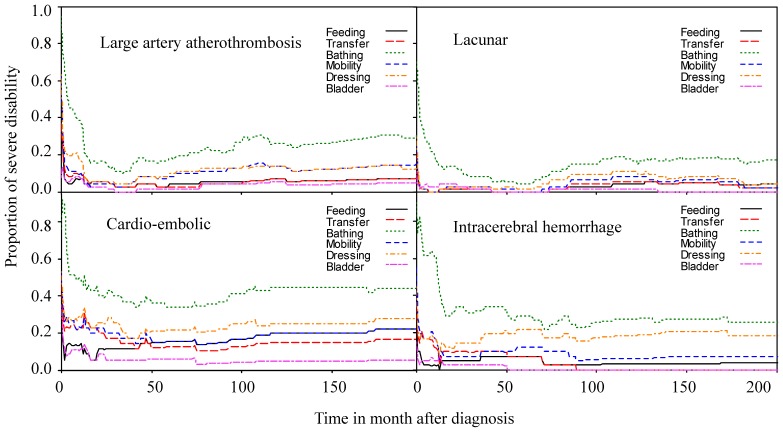
Dynamic changes in functional needs for patients with severe disabilities after stroke.

**Figure 3:**
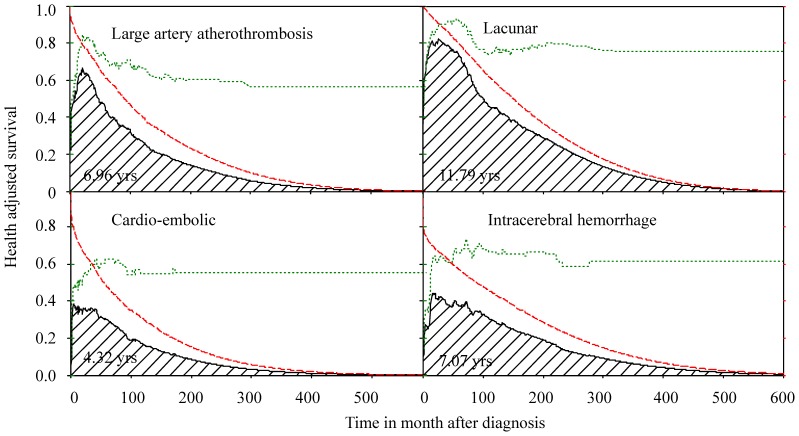
The lifetime health-adjusted survival of stroke patients. The survival probability (longdash line) multiplied by the proportion of patients with no disability (dotted line) over time after diagnosis results in the health-adjusted survival curve (solid line), which can be summed to estimate the expected life years without functional disabilities for stroke patients (shaded area).

The proportions of different physical functional disabilities among stroke patients with individual BI items (e.g., feeding, transfer, bathing, and so on) scored as zero were plotted against time after diagnosis (Figure 2), and the lifelong durations for the care needs of different functional items in BI are summarized in Table 2. The results show that patients with CEI required the longest duration of self-care and mobility assistance, with 3.37 (standard error of mean (SEM), 0.62) years for bathing, 1.89 (SEM, 0.54) years for toilet use, 1.57 (SEM, 0.43) years for mobility, and 0.49 (SEM, 0.27) years for bladder control. Patients with lacunar infarct appeared to have the least need for long term care for every BI item (Table 2). On average, the total life expectancy of stroke patients was composed of 8.35 (95% confidence intervals (CI), 7.88–8.83) years without disability, 0.86 (95% CI, 0.53–1.20) years with mild disability, 1.24 (95% CI, 0.94–1.60) years with moderate disability and 1.39 (95% CI, 1.07–1.65) years with severe disability. Figure 3 shows the disability-free durations for the four major subtypes of stroke. Patients with CEI suffered the longest period of severe disability (2.35 years) and the shortest life expectancy, followed by patients with ICH and LAA (Table 3). However, there is no statistical difference between the mean durations of CEI and ICH for the severe disability.

**Table 2:** Mean (standard error, in years) duration of subjects with different levels and types of functional disabilities, as measured by the Barthel Index (BI) and stratified by stroke subtypes.

Category		Infarct				ICH‡
		LAA*	Lacune	CE†	Others	
BI item	Score					
Feeding	10	9.55(0.30)	13.75(0.32)	6.16(0.50)	11.05(0.20)	10.35(0.47)
	5	0.32(0.16)	0.39(0.20)	0.40(0.26)	0.15(0.05)	0.67(0.34)
	0	0.63(0.20)	0.43(0.21)	1.29(0.45)	0.35(0.17)	0.42(0.29)
Transfer	15	7.99(0.36)	12.91(0.36)	5.04(0.53)	9.59(0.42)	9.27(0.59)
	10	0.87(0.30)	0.84(0.24)	0.78(0.34)	1.23(0.44)	0.83(0.37)
	5	0.85(0.26)	0.35(0.17)	0.76(0.38)	0.49(0.25)	0.90(0.45)
	0	0.63(0.20)	0.36(0.15)	1.29(0.43)	0.30(0.15)	0.41(0.16)
Grooming	5	9.04(0.29)	13.47(0.32)	5.70(0.54)	10.32(0.37)	8.95(0.68)
	0	1.48(0.34)	1.18(0.31)	2.14(0.61)	1.30(0.43)	2.46(0.62)
Toilet use	10	8.34(0.42)	13.13(0.35)	4.83(0.58)	9.96(0.44)	8.58(0.65)
	5	0.99(0.25)	0.64(0.22)	1.12(0.45)	0.86(0.27)	1.30(0.42)
	0	1.18(0.32)	0.73(0.24)	1.89(0.54)	0.81(0.29)	1.51(0.49)
Bathing	5	7.76(0.43)	12.30(0.40)	4.45(0.59)	9.20(0.51)	7.81(0.69)
	0	2.76(0.40)	2.30(0.36)	3.37(0.62)	2.44(0.52)	3.34(0.79)
Mobility	15	8.29(0.38)	12.42(12.41)	5.03(0.54)	8.89(0.56)	8.73(0.62)
	10	0.92(0.26)	1.35(0.30)	1.23(0.42)	1.56(0.56)	1.56(0.55)
	5	0.09(0.05)	0.26(0.21)	0.05(0.03)	0.17(0.08)	0.15(0.08)
	0	1.21(0.26)	0.52(0.19)	1.57(0.43)	0.99(0.41)	0.95(0.37)
Stairs	10	7.09(0.43)	11.87(0.44)	4.57(0.62)	7.99(0.76)	7.80(0.65)
	5	1.01(0.31)	0.62(0.18)	0.68(0.36)	0.48(0.27)	0.47(0.27)
	0	2.39(0.38)	2.06(0.39)	2.60(0.52)	3.20(0.64)	3.13(0.62)
Dressing	10	8.50(0.39)	12.59(0.38)	4.70(0.58)	9.39(0.45)	8.23(8.23)
	5	0.79(0.23)	1.20(0.30)	1.18(0.44)	1.29(0.38)	1.05(0.41)
	0	1.26(0.30)	0.78(0.23)	1.96(0.51)	0.96(0.29)	2.13(0.62)
Bowels	10	9.85(0.27)	14.18(0.25)	7.05(0.35)	11.37(0.16)	10.90(0.32)
	5	0.54(0.20)	0.35(0.19)	0.73(0.33)	0.08(0.04)	0.44(0.26)
	0	0.13(0.06)	0.07(0.04)	0.06(0.04)	0.17(0.12)	0.05(0.02)
Bladder	10	9.69(0.24)	13.84(0.26)	6.69(0.41)	11.16(0.24)	10.75(0.32)
	5	0.39(0.19)	0.60(0.27)	0.65(0.32)	0.17(0.08)	0.55(0.32)
	0	0.40(0.17)	0.14(0.06)	0.49(0.27)	0.39(0.23)	0.10(0.07)

* LAA: Large artery atherothrombosis;

† CE: Cardio-embolism;

‡ ICH: Intracerebral hemorrhage.

**Table 3:** Estimation of life expectancy (LE, 95% confidence interval (CI), in years), EYLL (expected years of life loss, with standard error of mean in parenthesis), mean lifelong duration (95% CI, in years) of each functional disability state as measured by the Barthel Index (BI) and EYLD (expected years of living with disability) stratified by different stroke subtypes.

Category of stroke	Infarct				ICH**
	LAA^| |^	Lacune	CE#	Others	
Average age	68.38	68.29	70.38	61.04	59.75
LE	10.50 (10.29–10.75)	14.66 (14.49–14.93)	7.85 (7.64–8.05)	11.67 (11.50–11.84)	11.37 (11.17–11.57)
EYLL	4.41(0.12)	1.69(0.11)	7.28(0.12)	7.62(0.09)	10.50±0.10
Years with no disability*	6.96 (6.13–7.86)	11.79 (10.97–12.46)	4.32 (3.37–5.57)	8.28 (6.94–9.12)	7.07 (5.48–8.23)
(% subjects)	(54%)	(73%)	(29%)	(61%)	(38%)
Years with mild disability†	0.91 (0.54–1.44)	0.48 (0.29–0.85)	0.23 (0.01–0.76)	1.06 (0.54–1.76)	1.15 (0.44–1.99)
(% subjects)	(10%)	(6%)	(4%)	(11%)	(17%)
Years with moderate disability‡	1.17 (0.63–1.89)	1.44 (0.83–2.20)	0.95 (0.32–1.62)	1.31 (0.74–2.22)	1.15 (0.36–1.88)
(% subjects)	(18%)	(14%)	(22%)	(13%)	(22%)
Years with severe disability§	1.46 (0.85–2.06)	0.95 (0.46–1.49)	2.35 (1.25–3.38)	1.02 (0.54–1.65)	2.00 (0.82–3.32)
(% subjects)	(18%)	(7%)	(45%)	(15%)	(23%)
EYLD	3.54	2.87	3.53	3.39	4.30

* no disability (BI = 100);

† mild disability (BI = 90–95);

‡ moderate disability (BI = 60–85);

§ severe disability (BI = ≤55);

| | LAA:Large artery atherothrombosis;

# CE: Cardio-embolism;

** ICH: Intracerebral hemorrhage.

## Discussions

While the Sullivan method has been used to integrate the weight of functional disabilities with survival in the general population to compute health expectancies [24], it has never been applied to an actual cohort of a particular illness with functional disabilities, such as stroke patients, because there are usually no life tables for such groups. We applied a novel semi-parametric method to extrapolate the survival function of stroke patients to lifetime under the assumption of constant excess hazard (or mortality rate), which solves this fundamental problem. The survival probability was then multiplied with the proportion of functional disabilities at each time *t* and summed up to estimate lifelong durations for different disabilities or needs for long term care (an example shown in Figure 3). Under ideal conditions, all the functional measurements (BI, cognition, and others) would be performed for all patients, including those with any pre-existing disability, who would then be followed longitudinally for the rest of their lives to obtain the durations for newly developed functional impairments and different long term care needs. However, such studies would require several decades to complete. We took an alternative approach to recruit cross-sectional, consecutive patients from stroke clinics and rehabilitation wards, and the data thus obtained can be used along with the survival function to estimate the lifelong duration, if it is a random sample and the sample size is over 50 [23]. We consecutively sampled patients from stroke clinics and rehabilitation wards and the number of patients was more than three times the above requirement to compensate for non-random sampling (Table 1). This study has following strengths: The NTUH Stroke Registry Cohort has been shown to be representative of Taiwanese stroke patients in general, if compared with all those hospitalized under NHI (Table 1); the follow-up period for this group was over 15 years, and physical functional disabilities were directly assessed on consecutive patients in both stroke clinics and rehabilitation wards. Therefore, we tentatively conclude that the durations of severe physical functional disabilities, which indicate the need for long-term care, are usually the longest in patients with CEI and ICH, while those of patients with lacunar infarct are the shortest (Table 2). In addition, the expected years of life lost are the highest in ICH patients because they have the lowest onset age. ICH patients also have the longest total duration spent with disability, or 4.30 years of EYLDs (Table 3).Such differential needs must thus be considered early in daily care and long term rehabilitation programs for stroke patients in order to improve their quality of life and survival. Such estimations may also be extended to other functional disabilities (e.g., cognition, speech, or vision) to estimate other long-term care needs, and will be useful not only for preparing the patient’s family for the prognosis, but also for national resource planning with regard to long-term care after taking the different incidences of each subtype of stroke into account.

Among the various BI items, the most common need for care assistance was with bathing, and the need for this totaled 2.76, 2.30, 3.37, and 3.34 years in patients with LAA, lacunar infarct, CEI, and ICH, respectively (Table 2). Dynamic changes in the need for assistance by patients with severe disabilities appeared to decrease within the first two years for all subtypes of stroke (Figure 2), but they increased after a nadir, probably because of aging and multiple co-morbidities. Patients with lacunar infarct recovered quickly and suffer the least for most items (Figure 2 and Table 2). The results indicate that CEI patients suffered the greatest physical functional disabilities compared to other patients with ischemic stroke, because these often had coexisting heart problems (Table 1), such as ischemic heart diseases, heart failures, or arrhythmia, and might have difficulty swallowing. In total, patients with CEI would be expected to require longer duration of self-care and mobility assistance throughout their lifetimes, with 1.29 years for feeding, 0.49 years for bladder control, 1.29 years for transfer, and 1.57 years for mobility (Table 2). Patients with intracranial hemorrhages also suffered from such functional impairments with a similar magnitude.

### Potential Limitations

This study has the following limitations: First, when we recruited our subjects from both stroke clinics and rehabilitation wards, the most severe patients who were unable to communicate with the clinicians, e.g., patients with very poor cognition and/or aphasia, were not included. Although the most severely disabled patients quickly died, we may still have underestimated the true proportion of disability [8]–[10], and the results of this study are to be regarded as a lower bound for planning the service needs related to long-term care for different subtypes of stroke. Second, the 130 patients from the rehabilitation wards were assessed two times for their BI, once in the hospital and the second time one year after diagnosis via a telephone interview. The telephone assessment of stroke disability using the BI scale is acceptable in reliability in comparison to direct face-to-face assessments in clinically stable patients with stroke [25], and these patients might be over-represented in this study. However, because these patients made up only 11% of our patients and the repeated assessments were performed almost one year apart, our estimation is probably not too biased. To validate the above claim, we conducted a sensitivity analysis by including only the data from the first assessment, namely, n = 1,162, and the results are almost the same, with slightly wider confidence limits because of the smaller sample size, as shown in the Table S1. Finally, functional disabilities are heavily influenced by age and comorbidities, such as rheumatoid arthritis [26], Alzheimer’s disease [27], and so on. While stroke usually occur in old age, or above 65 year-old, there are wide variations, as indicated by the large standard deviations (Table 1), and some people may have a stroke when they are significantly younger than this [28]–[30]. Stratifications by age and/or major co-morbidities would thus help achieve a more accurate estimation. It is recommended that future studies regularly assess stroke patients with regard to various types of functional disabilities, as well as carry out cognitive and emotional assessments, in order to collect more detailed and accurate data, so that a better determination of the long-term care needs of different age groups can be obtained.

### Conclusions

By analyzing data directly obtained from stroke patients, this study found that CEI and ICH patients suffer the longest duration of different physical functional disabilities related to self-care, followed by those with LAA infarct, whereas patients with lacunar infarct suffer for the shortest period of time. Assistance in bathing appears to be the most common need for care. The estimated results could be used by all stakeholders to reach a consensus on related health policies. Most importantly, this study demonstrates that the integration of a survival function from a cohort with a consecutive random sample for direct measurements of functional disability items and levels could provide valuable information (or the lower bound) for the determination of long-term care needs, which could lead to better planning for long-term care by health insurers and government agencies.

## Supporting Information

Table S1 Estimation of life expectancy (LE, 95% confidence interval (CI), in years), EYLL (expected years of life loss, with standard error of mean in parenthesis), mean lifelong duration (95% CI, in years (Yrs)) of each functional disability state as measured by the Barthel Index (BI) and EYLD (expected years of living with disability) stratified by different stroke subtypes, of which only data of first assessment were included (n = 1,162).
(DOCX)

Details of the formula S1 (DOCX)

## References

[1] World Health Organization (2008) Fact Sheet No. 310: The top ten causes of death. Geneva: World Health Organization.

[2] Go AS, Mozaffarian D, Roger VL, Benjamin EJ, Berry JD, et al (2013) Heart disease and stroke statistics–2013 update: a report from the American Heart Association. Circulation 127: e6–e245. 23239837 10.1161/CIR.0b013e31828124ad PMC5408511

[3] Centers for Disease Control and Prevention (CDC) (2007) Outpatient rehabilitation among stroke survivors–21 states and the District of Columbia, 2005. MMWR 56: 504–507. 17522589

[4] Adams HP Jr, Bendixen BH, Kappelle LJ, Biller J, Love BB, et al (1993) Classification of subtype of acute ischemic stroke. Definitions for use in a multicenter clinical trial. TOAST. Trial of Org 10172 in Acute Stroke Treatment. Stroke 24: 35–41. 7678184 10.1161/01.str.24.1.35

[5] Turin TC, Kita Y, Rumana N, Nakamura Y, Takashima N, et al (2010) Ischemic stroke subtypes in a Japanese population: Takashima Stroke Registry, 1988–2004. Stroke 41: 1871–1876. 20689083 10.1161/STROKEAHA.110.581033

[6] Kolominsky-Rabas PL, Weber M, Gefeller O, Neundoerfer B, Heuschmann PU (2001) Epidemiology of ischemic stroke subtypes according to TOAST criteria: incidence, recurrence, and long-term survival in ischemic stroke subtypes: a population-based study. Stroke 32: 2735–2740. 11739965 10.1161/hs1201.100209

[7] Yip PK, Jeng JS, Lee TK, Chang YC, Huang ZS, et al (1997) Subtypes of ischemic stroke: a hospital-based stroke registry in Taiwan (SCAN-IV). Stroke 28: 2507–2512. 9412641 10.1161/01.str.28.12.2507

[8] Kimura K, Kazui S, Minematsu K, Yamaguchi T (2004) Japan Multicenter Stroke Investigator's Collaboration (2004) Analysis of 16,922 patients with acute ischemic stroke and transient ischemic attack in Japan. A hospital-based prospective registration study. Cerebrovasc Dis 18: 47–56. 15178987 10.1159/000078749

[9] Grau AJ, Weimar C, Buggle F, Heinrich A, Goertler M, et al (2001) Risk factors, outcome, and treatment in subtypes of ischemic stroke: the German stroke data bank. Stroke 32: 2559–2566. 11692017 10.1161/hs1101.098524

[10] Petty GW, Brown RD Jr, Whisnant JP, Sicks JD, O'Fallon WM, et al (2000) Ischemic stroke subtypes: a population-based study of functional outcome, survival, and recurrence. Stroke 31: 1062–1068. 10797166 10.1161/01.str.31.5.1062

[11] Hsueh IP, Lee MM, Hsieh CL (2001) Psychometric characteristics of the Barthel activities of daily living index in stroke patients. J Formos Med Assoc 100: 526–532. 11678002

[12] Hsueh IP, Lin JH, Jeng JS, Hsieh CL (2002) Comparison of the psychometric characteristics of the functional independence measure, 5 item Barthel index, and 10 item Barthel index in patients with stroke. J Neurol Neurosurg Psychiatry 73: 188–190. 12122181 10.1136/jnnp.73.2.188 PMC1737984

[13] Lee HY, Hwang JS, Jeng JS, Wang JD (2010) Quality-Adjusted Life Expectancy (QALE) and loss of QALE for patients with ischemic stroke and intracerebral hemorrhage: A 13-year follow-up. Stroke 41: 739–744. 20150543 10.1161/STROKEAHA.109.573543

[14] Quinn TJ, Langhorne P, Stott DJ (2011) Barthel index for stroke trials: development, properties, and application. Stroke 42: 1146–1151. 21372310 10.1161/STROKEAHA.110.598540

[15] Granger CV, Dewis LS, Peters NC, Sherwood CC, Barrett JE (1979) Stroke rehabilitation: analysis of repeated Barthel index measures. Arch Phys Med Rehabil 60: 14–17. 420565

[16] Uyttenboogaart M, Stewart RE, Vroomen PC, De Keyser J, Luijckx GJ (2005) Optimizing cutoff scores for the Barthel index and the modified Rankin scale for defining outcome in acute stroke trials. Stroke 36: 1984–1987. 16081854 10.1161/01.STR.0000177872.87960.61

[17] Lee ET, Wang JW (2009) Nonparametric Methods of estimating survival functions. In: Lee ET, and Wang JW, eds. Statistical methods for survival data analysis. 3rd Ed. New York: John Wiley & Sons. 64–105.

[18] Fang CT, Chang YY, Hsu HM, Twu SJ, Chen KT, et al (2007) Life expectancy of patients with newly-diagnosed HIV infection in the era of highly active antiretroviral therapy. QJM 100: 97–105. 17277317 10.1093/qjmed/hcl141

[19] iSQoL software. Available: [http://www.stat.sinica.edu.tw/jshwang]. Accessed 17April, 2013.

[20] Chu PC, Wang JD, Hwang JS, Chang YY (2008) Estimation of life expectancy and the expected years of life lost in patients with major cancers: extrapolation of survival curves under high-censored rates. Value Health 11: 1102–1109. 18489497 10.1111/j.1524-4733.2008.00350.x

[21] Liu PH, Wang JD, Keating NL (2013) Expected years of life lost for six potentially preventable cancers in the United States. Prev Med 56: 309–313. 23428566 10.1016/j.ypmed.2013.02.003

[22] Hwang JS, Wang JD (1999) Monte Carlo estimation of extrapolation of quality-adjusted survival for follow-up studies. Stat Med 18: 1627–1640. 10407234 10.1002/(sici)1097-0258(19990715)18:13<1627::aid-sim159>3.0.co;2-d

[23] Hwang JS, Tsauo JY, Wang JD (1996) Estimation of expected quality adjusted survival by cross sectional survey. Stat Med 15: 93–102. 8614748 10.1002/(SICI)1097-0258(19960115)15:1<93::AID-SIM155>3.0.CO;2-2

[24] Health Expectancy Calculation by the Sullivan Method: A Practical Guide 2007. Available: http://reves.site.ined.fr/en/resources/computation_online/sullivan/. Accessed 17 April 2013.

[25] Della Pietra GL, Savio K, Oddone E, Reggiani M, Monaco F, et al (2011) Validity and reliability of the Barthel index administered by telephone. Stroke 42: 2077–2079. 21527755 10.1161/STROKEAHA.111.613521

[26] Shin SY (2012) The relationship between cognitive and physical function in older adults with rheumatoid arthritis: a literature review. J Gerontol Nurs 38: 33–42. 22897128 10.3928/00989134-20120807-03

[27] Castellani RJ, Rolston RK, Smith MA (2010) Alzheimer disease. Dis Mon 56: 484–546. 20831921 10.1016/j.disamonth.2010.06.001 PMC2941917

[28] Spengos K, Vemmos K (2010) Risk factors, etiology, and outcome of first-ever ischemic stroke in young adults aged 15 to 45 - the Athens young stroke registry. Eur J Neurol 17: 1358–1364. 20482604 10.1111/j.1468-1331.2010.03065.x

[29] Putaala J, Curtze S, Hiltunen S, Tolppanen H, Kaste M, et al (2009) Causes of death and predictors of 5-year mortality in young adults after first-ever ischemic stroke: the Helsinki Young Stroke Registry. Stroke 40: 2698–2703. 19590052 10.1161/STROKEAHA.109.554998

[30] Putaala J, Metso AJ, Metso TM, Konkola N, Kraemer Y, et al (2009) Analysis of 1008 consecutive patients aged 15 to 49 with first-ever ischemic stroke: the Helsinki young stroke registry. Stroke 40: 1195–1203. 19246709 10.1161/STROKEAHA.108.529883

