# Supplementary material for: Estimation of the Long-Term Care Needs of Stroke Patients by Integrating Functional Disability and Survival
Source: PLoS One. 2013 Oct 4;8(10):e75605. doi: 10.1371/journal.pone.0075605 (PMC3790845; doi:10.1371/journal.pone.0075605)
Supplement: Table S1 — Estimation of life expectancy (LE, 95% confidence interval (CI), in years), EYLL (expected years of life loss, with standard error of mean in parenthesis), mean lifelong duration (95% CI, in years (Yrs)) of each functional disability state as measured by the Barthel Index (BI) and EYLD (expected years of living with disability) stratified by different stroke subtypes, of which only data of first assessment were included (n = 1,162). (DOCX) [file pone.0075605.s001.docx]

Table S1. Estimation of life expectancy (LE, 95% confidence interval (CI), in years), EYLL (expected years of life loss, with standard error of mean in parenthesis), mean lifelong duration (95% CI, in years(Yrs)) of each functional disability state as measured by the Barthel Index (BI) and EYLD (expected years of living with disability) stratified by different stroke subtypes, of which only data of first assessment were included (n=1,162).

| Category of stroke | **Infarct** | | | | **ICH* *** |
| --- | --- | --- | --- | --- | --- |
|  | **LAA^\| \|^** | **Lacune** | **CE^#^** | **Others** |  |
| Case number | 260 | 339 | 152 | 251 | 160 |
| Average age | 68.48 | 68.44 | 70.28 | 60.79 | 60.41 |
| LE | 10.54 (10.32-10.70) | 14.55 (14.41-14.74) | 7.85 (7.58-8.11) | 11.67 (11.48-11.84) | 11.42 (11.26-11.59) |
| EYLL | 4.38(0.11) | 1.79(0.09) | 7.21(0.12) | 7.63(0.09) | 10.45(0.10) |
| Years with no disability^*^ | 7.06 (6.10-8.06) | 11.80 (11.08-12.59) | 4.50 (3.38-5.66) | 8.39 (7.42-9.46) | 7.26 (5.66-8.33) |
| (% subjects) | (55%) | (74%) | (29%) | (63%) | (35%) |
| Years with mild disability^†^ | 0.93 (0.41-1.49) | 0.48 (0.18-0.82) | 0.14 (0.00-0.83) | 1.02 (0.36-1.79) | 1.10 (0.45-2.04) |
| (% subjects) | (9%) | (6%) | (4%) | (10%) | (17%) |
| Years with moderate disability^‡^ | 1.15 (0.53-1.60) | 1.34 (0.67-2.06) | 0.93 (0.27-1.79) | 1.28 (0.66-2.06) | 1.25 (0.61-21.72) |
| (% subjects) | (18%) | (13%) | (22%) | (13%) | (22%) |
| Years with severe disability^§^ | 1.40 (0.88-2.12) | 0.93 (0.43-1.69) | 2.28 (1.23-3.26) | 0.98 (0.33-1.57) | 1.81 (0.87-3.08) |
| (% subjects) | (18%) | (7%) | (45%) | (14%) | (26%) |
| EYLD | 3.48 | 2.75 | 3.35 | 3.28 | 4.16 |

*no disability (BI= 100); ^†^ mild disability (BI= 90-95); ^‡^ moderate disability (BI=60-85); ^§^severe disability (BI= ≤55);

^| |^ LAA:Large artery atherothrombosis; ^#^ CE: Cardio-embolism; * *ICH: Intracerebral hemorrhage
