# Supplementary material for: Estimation of the Long-Term Care Needs of Stroke Patients by Integrating Functional Disability and Survival
Source: PLoS One. 2013 Oct 4;8(10):e75605. doi: 10.1371/journal.pone.0075605 (PMC3790845; doi:10.1371/journal.pone.0075605)
Supplement: Details of the formula S1 — (DOCX) [file pone.0075605.s002.docx]

**Details of the formula. S1**

Since subjects’ health states often vary over time, we can define a health status function as if the health status is $H_{j}$for subject *i* at time *t*; otherwise 0. The *i*-th subject’s lifelong duration with health status $H_{j}$ from onset to death is (Reference 1). The mean lifelong duration of a population with health status $H_{j}$is

where *N* is the population size. The integrand can be further represented as

where *G*(*t*) is the subpopulation of subjects still alive at time *t*, *M*(*t*) is the size of *G*(*t*) and $S(t)=M(t)/N$ is the survival function of the population with the specific condition.

**References**

1. MurrayC J L, Salomon JA, Mathers CD, Lopez AD. (2002) Summary measures of population health: Concepts, ethics, measurement and applications. Geneva: World Health Organization.
